# Thinking Together; A Social Care Learning Community, ‘10 Minutes of Britney’ and Flourishing Lives

**DOI:** 10.1111/jar.70281

**Published:** 2026-07-07

**Authors:** Sara Ryan, Siabhainn Russell

**Affiliations:** ^1^ Manchester Metropolitan University Manchester UK; ^2^ University of Oxford Oxford UK

## Abstract

**Background:**

People with intellectual disabilities continue to experience poor support despite considerable research evidence about what could be improved.

**Methods:**

An online learning community of people with intellectual disabilities, carers, and social care staff, grounded in Sen's Capability Approach, explored how good support can be delivered in practice. Six sessions covered topics including confidence, belonging and thinking ahead.

**Results:**

Thinking together generated ‘lightbulb moments’, most notably the realisation seemingly trivial preferences, like listening to Britney Spears, can hold profound personal value, yet are overlooked in formal care‐planning. Lay team members went on tour with the findings, producing additional examples of low‐cost activities termed ‘10 Minutes of Britney’.

**Conclusion:**

Social care practice could be improved by systematically recognising and enabling such everyday capabilities, particularly within resource‐constrained, austerity influenced service environments. It is important that these capabilities remain relevant to us all, rather than a distinct intervention for people with intellectual disabilities.

## Lay Summary

- It is important to understand that little things which may seem unimportant can matter a lot to people.
- These things can be left out of care plans because they seem so small, even though they are what people value and enjoy.
- Recognising these small things and supporting people to be able to do them is often cheap and easy to organise.
- An online learning community is a good way of thinking together about improving social care support.

The Flourishing Lives project aimed to try to move beyond the inertia surrounding improvements in the lives of people with intellectual disabilities using Amartya Sen's (2009) Capability Approach (CA). We have a considerable research evidence‐base and associated knowledge about what *could* be improved, yet people with intellectual disabilities continue to experience impoverished lives and health inequalities. Premature mortality rates remain untouched, employment rates remain low, people are unlikely to be supported to form relationships or have families of their own, and often continue to live with ageing parents (McCarthy et al. 2022; Taubner et al. 2022; Ryan et al. 2024). Johnson et al. (2010, 127) suggest a Capabilities Approach offers exciting potential for working with people with learning disabilities as it moves beyond practices associated with person‐centred care to ‘more spiritual and emotional’ realms that are not often considered in the lives of people with intellectual disabilities. We designed a project with two stages. Stage 1 involved focus groups and interviews with people with intellectual disabilities and family carers exploring what was important to them. An overarching theme of ‘doing what you love and growing’ alongside three subordinate themes; ‘choice, opportunity and empowerment’, ‘being out in the world’ and ‘lowered expectations and static lives’ were identified (Ryan and O'Brien 2024). This paper reports on Stage 2 of the project which set out to explore how people with intellectual disabilities can be better supported to lead good lives by creating a learning community of support workers, support managers, people with learning disabilities, family carers and academics.

The theoretical foundations of CA are elaborated in the first paper and only briefly summarised here. For Sen (2009), health and wellbeing fundamentally depend on the freedoms and opportunities—the capabilities—people have to do things they value. CA has been influential in developing understandings of individual wellbeing to reduce social injustices and has a key underpinning principle that everyone is worthy of respect (Sen 2009). This perspective is particularly salient in the context of under‐resourced social care systems under austerity conditions (Koch and James 2022; Mos and Reckers‐Droog 2024).

## 1 Learning Communities

A learning community or ‘community of practice’ is a partnership created by people who care about the same issue, to learn from and with each other. Learning communities as a mechanism of sharing knowledge and tools have evolved and been prominent in the UK since the 1980s. Early examples were geographically focused with prominent examples revolving around ‘learning cities’ and ‘learning regions’ (see, for example Longworth 2012). The geographical focus lessened over time and, instead of predominantly focusing on knowledge exchange, learning communities began to be used to provide members with support and a means of professional development. This type of learning community has often been used in educational settings (Kilpatrick et al. 2003; Ballangrud and Aas 2022). Learning communities, involving professionals, patients, and members of the business and/or charity sector, have also been used in healthcare to assist with achieving sustainable, consistent improvements in practice and service provision across a range of services. Examples include developing improved services for patients suffering with inflammatory bowel disease (Crohn's and Colitis UK n.d.) and increasing HIV (Human Immunodeficiency Virus) awareness and testing in marginalised African and Caribbean communities (Hardie et al. 2022). There is sparse evidence about the use of learning communities in social care practice (see, for example, Cook‐Craig and Sabah 2009; Murty et al. 2012).

For Wenger (1998), learning community involvement features a combination of participation and reification; interaction and the production of artefacts like tools, concepts and theory. These resources can be further shared with wider communities of practice resulting in tools or knowledge improving practice or skills within the profession (Townley 2020). Learning communities can also be sources of innovation (Nicolini et al. 2022) and are held to be a process, rather than static, a space where people come together, albeit sometimes only occasionally (Pyrko et al. 2016). Tensions exist around whether a learning community can be ‘set up’, as they were originally held to be spontaneous groupings of people with investment of identity in the particular social context (Amin and Roberts 2008). For Iverson and McPhee (2008), for example, learning communities cannot be set up as a formal team and it is more important to pay attention to the nuances of lived practice, a position subsequently interpreted as ‘knowing in practice’ with knowledge relegated to the potentiality to act (Pyrko et al. 2016). At the heart of a learning community is a commitment to thinking together without which the learning community would not exist;

As soon as thinking together at the heart of the community stops, it will quickly begin to lose its rhythm and vibrancy (or it may never come into life in the first place). (Pyrko et al. 2016, 403)

Learning community members guide each other through understandings of the issue under focus and in doing so, indirectly share tacit knowledge and learning (Wenger et al. 2011). It is this sharing that enriches the community and generates alternative understandings and knowledge which are greater than the sum of the individual members' knowledge. Indeed, it is argued these communities involve participants renegotiating the meaning of personal experiences and shifts in identities (Pyrko et al. 2016). Four propositions—the importance of commitment to change, interactional nuance, thinking together and contributions of members—have been suggested which underline the importance of commitment to change, and a focus on the interactional nuance generated in these spaces rather than didactic teaching. That is, the centrality of thinking together and the different contributions of members to that thinking (Pyrko et al. 2016).

The Flourishing Lives learning community brought together people with intellectual disabilities, family carers, support workers and managers, a senior provider manager and academics providing an innovative space within social care research to discuss issues around how people with intellectual disabilities can be better supported to lead good lives. It was conceptualised as a practical tool to think together through enduring problems in social care support, drawing on the findings of the qualitative fieldwork in Stage 1. We do not need to re‐rehearse the enduringly poor conditions people with intellectual disabilities experience in the UK, as a weighty evidence‐base demonstrates this (see, for example, Emerson and Hatton 2008; Brusch 2017; Malli et al. 2018; Ramsey et al. 2022). A key issue is, however, the acceptability of poor practice and widely held assumptions that the conditions under which people with intellectual disabilities live are good enough. Social care in this area tends to be evaluated by negative freedoms, that is, freedoms from hunger, poor housing and harm rather than positive freedoms; living a flourishing and growing life with opportunities and possibilities. This means people with intellectual disabilities often lead static and limited lives (Ryan 2025). Using a Capability Approach allowed us to focus on what it is that people with intellectual disabilities value doing and how they may be supported to have the freedom and support to do these activities.

## 2 Methods

The Flourishing Lives Learning Community was originally planned to be held face‐to‐face with three meetings across 11 months; 1.5 days, half a day, and 1 day respectively. This design was drawn from a study exploring how frontline teams engage with patient‐centred quality improvement (Locock et al. 2021). The Covid‐19 pandemic generated a requirement for methodological innovation and the Learning Community moved online (Richardson et al. 2021; Mikulak et al. 2023). On reflection, this move had some benefits. It allowed flexibility around attendance for support workers, removing travel time and concentrated sessions. Some participants, for example, dealt with interruptions during sessions. The growing momentum and coherence would have been harder to establish with longer gaps between meetings. For participants with learning disabilities, online research can involve additional considerations (Mikulak et al. 2023); however in this instance, the participation of people with intellectual disabilities was part of longer‐term involvement in research with established relationships with the research team.

Social care staff participants were recruited via the Gr8 Support Movement led by third sector organisation, Paradigm, social media platforms and the research team networks. Three people with intellectual disabilities and two family carers who were part of the research team also joined the community.

The Learning Community met online via Teams or Zoom depending on the preferences of members every 6 weeks, focusing on a different topic based on the findings of Stage 1 of the project. Each session was repeated two times led by two experienced facilitators, one with intellectual disabilities, to enable flexible participation. A third session was led by the research team where necessary. The sessions lasted between 90 and 110 min. Session 1 introduced participants to each other and the aims and objectives of the project, Session 2 focused on belonging, Session 3 on confidence, Session 4 on thinking ahead and Session 5 introduced CA. Session 6 involved drawing together the various discussions and thinking about the content and shape of the project resources.

**FIGURE 1:**
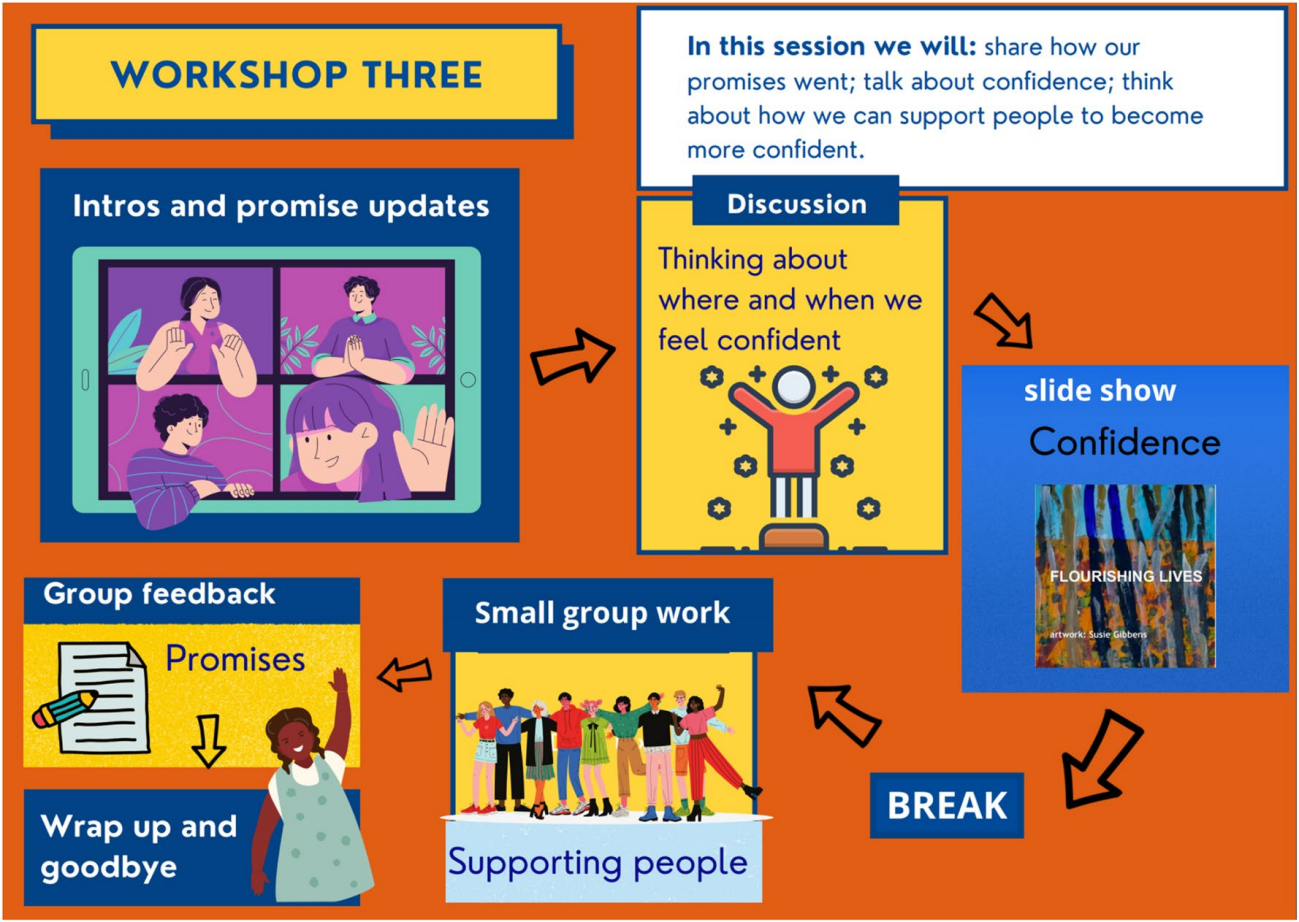
Example workshop template.

Each session was structured using a template which also acted as an orienteering device (see Figure 1). Promises by social care staff participants were introduced to generate continuity across the sessions and often involved tiny tweaks in practice, such as introducing a small change in the route someone walked daily with a view to expanding their engagement with their neighbourhood. Participants were asked to first feedback on their promise of a small change to practice. The topic of the session was introduced using data extracts from Stage 1 of the project to prompt discussion (Ryan and O'Brien 2024).

Participants were asked about their own experiences of the topic, before broadening the discussion out to how the topic related to supporting other people, or how participants with intellectual disabilities could be better supported in this area.

Twenty four participants joined the learning community from across England. The flexible design of the workshops and changing of jobs during the project meant that there was shifting attendance as illustrated in Table 1. Some staff attended during their working day which meant they sometimes left the workshop to take calls or deal with emerging issues. Perhaps because of the commitment of participants to improving support for people with learning disabilities, the composition of the groups did not appear to affect the richness of the discussion. The facilitators in effect worked as non‐judgemental peer mentors (Pyrko et al. 2016) and created a space in which participants appeared to feel comfortable and engaged. Informal online drop‐ins held at the mid‐point between the six weekly sessions had varying attendance, creating space for people to check in briefly or longer, to say hello and feel connected to the community.

**TABLE 1:** Participant breakdown per workshop.

Session	A	B	C	Total
1	1 family carer	1 person with ID	2 support workers	26
	3 support workers	3 support workers	2 support managers	
	2 support managers	1 support manager	2 researchers	
	1 senior manager	2 researchers		
	2 researchers	1 facilitator with ID		
	1 facilitator with ID	1 facilitator		
	1 facilitator			
2	1 person with ID	4 support workers		18
	1 family carer	2 researchers		
	4 support workers	1 facilitator with ID		
	2 researchers	1 facilitator		
	1 facilitator with ID			
	1 facilitator			
3	1 person with ID	1 person with ID	1 person with ID	23
	1 support manager	1 family carer	1 family carer	
	2 researchers	5 support workers	manager	
	1 facilitator with ID	1 support manager	2 researchers	
	1 facilitator	2 researchers		
		1 facilitator with ID		
		1 facilitator		
4	1 person with ID	5 support workers		23
	6 support workers	2 support managers		
	2 researchers	1 senior manager		
	1 facilitator with ID	2 researchers		
	1 facilitator	1 facilitator with ID		
		1 facilitator		
5	1 family carer	1 person with ID	1 person with ID	24
	1 support worker	1 family carer	1 family carer	
	2 support managers	3 support workers	1 support worker	
	1 senior manager	2 academics	1 support manager	
	2 researchers	1 facilitator with ID	2 researchers	
	1 facilitator with ID	1 facilitator		
	1 facilitator			
6	2 people with ID	1 person with ID		20
	2 family carers	2 support workers		
	3 support workers	2 support managers		
	2 researchers	2 researchers		
	1 facilitator with ID	1 facilitator with ID		
	1 facilitator	1 facilitator		

Ethics approval was gained from the Manchester Metropolitan University ethics committee. Easy read information and consent forms were shared with potential participants and pre‐session meetings were offered to discuss the learning community and answer any questions.

## 3 Analysis

The workshops were audio and video recorded and loosely transcribed by a notetaker. The learning community was effectively a structured engagement programme comprising participants who were committed to thinking together to work through intractable problems. To this end, the data were carefully read, and recordings played to identify what and how community members contributed and interacted with each other, what sections stood out in terms of consensus, or where participants' expressed surprise, or became animated. Pyrko et al.'s (2016) proposition framework of the importance of commitment to change, interactional nuance, thinking together and contributions of members, was used as a framework to support the exploration and organisation of data.

## 4 Findings

Extended data extracts are presented under each proposition to illustrate the interactional nuance and how thinking together developed across the learning community sessions, culminating in new understandings of, and innovative practices in, supporting people to lead good lives.

### 4.1 Commitment to Change

In Session 1 participants were asked what their aim was in supporting people with intellectual disabilities. The discussion underlined shared interests among participants regardless of their different roles. The following extract involves an exchange between a support worker, family carer and facilitator:

Joe (Support worker): *Um, I want to better their lives, for the time I*'*m with them, I want to support them, I want them to achieve as much independence as they can. Um, just the right support, honestly. Just the right help that they require and as long as they*'*re happy, that*'*s the main thing. […] Seriously, a lot of my members they talk about happiness a lot of the time, especially… ah I can*'*t say his name but he literally never gets sad. I ask them as well, especially if they*'*re non verbal, I*'*ll ask them how they*'*re feeling, there*'*s always a way to communicate with them, I just want them to be happy and want them to have as much independence as possible and support them when it*'*s needed*.

Bea (family carer): *Can I just say that has literally… I was a little bit nervous about today because I thought I might get a little bit emotional, and I wasn*'*t sure quite how to handle it and that*'*s done it. So… [laughs]*

Mel (facilitator): *Positive emotion in response to Joe because you talk in such a human way about supporting someone, isn*'*t it Bea?*

Bea: *Well it*'*s my son you are talking about, I mean it*'*s not my son you are supporting Joe, but as a parent that*'*s what you hope for but you are never sure that*'*s what you*'*re going to get*.

This extract captures the commitment Joe described around supporting people with intellectual disabilities to lead good lives, and his contribution generated an emotional response from Bea. This emotion was picked up by the facilitators, who distilled the strength of his comments down to the humanity she felt he demonstrated. That is, simply asking people how they are feeling and supporting them to be as independent as necessary, regardless of whether they can verbally articulate their feelings. Bea made the discussion personal to her experiences by reflecting on how Joe could be talking about her son with intellectual disabilities and his commentary was what she would hope for in relation to the support her son may receive in the future. The power of this exchange in the first session, particularly given the uncertainties and precariousness around social care support for people with intellectual disabilities, underlines the commitment of participants to social change and improving the support for people with intellectual disabilities. This level of engagement, openness and commitment was consistent across sessions.

## 5 Interactional Nuance

The following extract is from Session 4 which focused on thinking ahead. Participants were invited to share examples of thinking ahead over the past year, describe the types of plans they made, and consider how they might help the person they work with think ahead. This interaction demonstrates how participants collaboratively teased out the distinction between thinking ahead and planning ahead, co‐constructing and refining their understanding through shared reflection;

Viv (Support worker): *and another thing I do a lot of with working shifts is planning, you know roughly what we're going to eat meals. I'm a, I love cooking, so I try a few new recipes every week. So I sit down with me books and plan what we're having and so I am a bit of a planner, but just every day*.

Mel (Facilitator): *Yeah*

Viv: *You're planning all the time and you don*'*t realise it do you?*

Mel: *I think so, yeah. Is there a difference, and this just occurred to me, I keep saying* “*planning ahead” but the words in, that came out as a theme was* “*thinking ahead”. Is there a difference between thinking ahead and planning ahead?*

Nat (Support Manager): *You have to go through the process of thinking, then be able to go through the process of planning I guess, so thinking kind of becomes, and then thinking through the whole sort of process as well*.

Ang (Support worker): *Planning is the doing of it isn*'*t it?*

Mel: *It is, isn't it?*

Ang: *Because I think about doing a lot of stuff that I never do, so [laughter] whereas planning is completely different, those are things I'll probably do, but just thinking I probably won't*.

This exchange demonstrates how learning communities can foster spaces for reflection and sustained engagement with ideas and thoughts that may otherwise remain implicit or unnoticed. As Mel said ‘it has just occurred to me’ highlighting how the distinction between thinking ahead and planning ahead was emergent. The space in this context enabled participants to generate what Geertz (1973) terms ‘thick description’, drawing connections between their personal experiences, collective sense‐making within the community and the potential implications for their future care support practices.

## 6 Thinking Together

Due in part to the facilitators' experience, thinking together emerged as a consistent and evolving feature of the workshops, and this developed as participants got to know each other. The extract below, from Session 2 (focused on belonging), illustrates the fluidity of collaborative thinking, or thinking together in practice. Mel deliberately shifted the session's pace by inviting participants to call out their thoughts. She began by gathering initial descriptions of feelings associated with belonging, then enriched the discussion by sharing personal details from her own experience. This prompted Pen to offer a concrete example of belonging, which in turn elicited further reflection from Nat, who affirmed the emerging list as a “good list.”

Liz (Facilitator with intellectual disabilities): *What does it feel like to belong somewhere?*

Mel: *So think about those places or those people you*'*re with where you go* ‘*oh god, I can relax, I feel I belong, I*'*m accepted for who I am, it just fits in lots of different ways*’ *What are the feelings you get from that? Just yell out, shall we just let people yell out Liz? Shall we break the rules and just get it out, go on!*

Nat: *Feel valued and appreciated*.

Mel: *Yep*.

Pen (Support worker): *Feel relaxed and comfortable*.

Mel: *Mmmm*.

Research team member: *Peaceful*

Liz (Support worker): *Love*

Jan (Support worker): *Happy*

Mel: *Pardon*

Jan: *Happy*.

Mel: *Happy, yeah. For me it*'*s being totally accepted as well, I*'*m a bit of a loud mouth, I just feel totally accepted, just oh, that*'*s Mel, that*'*s OK and that*'*s nice to feel*.

Nat: *Mmmm*.

Liz: *And wanted*.

Mel: *Wanted*.

Pen: *To be involved, I can remember when we was walking down the street one day and a neighbour in the neighbourhood give the lady I support some flowers out of her garden and you know, had a little chat with her, which was lovely*.

Mel: *Oooh, that gave me goosebumps that, that*'*s real belonging isn*'*t it? It*'*s about community, being seen and valued isn*'*t it?*

Pen: *Yeah*.

Nat: *Respected*.

Mel: *I think I feel equal as well, I don*'*t mean equal as in the same, but equal in that in [activity], none of us are perfect at it, we*'*re all a bit shoddy at times, but we*'*re all equal and that*'*s or just [inaudible] and doing thing. So, we, so far, we*'*ve got people feeling valued, appreciated, relaxed, comfortable, peaceful, loved, happy, accepted, wanted, involved, equal and respected*.

Nat: *Good list*

Further examples of thinking together included participants describing how the discussion prompted their thinking, in effect, sharing their workings out in real time. Jos, for example, reflected on her concerns as a new support worker. She shared the example of watching the television with the person she supported and being told by a senior staff member to clean the kitchen; ‘So sometimes what I think, just hearing the discussion now is, are we empowering people and saying it is ok to do things like sitting down, getting to know and becoming friends with the people you're supporting as well. You know, I was just thinking that when I was listening.’

Jos's reflections highlight a persistent tension in social care for people with intellectual disabilities: the frequent overshadowing of belonging, companionship, and meaningful social interaction by an overriding emphasis on procedural compliance and task‐oriented routines. When relational dimensions such as confidence, belonging, and thinking ahead are marginalised or overlooked, people's lives are inevitably constrained, and lacking in opportunities and possibilities.

## 7 Contributions of Members

**FIGURE 2:**
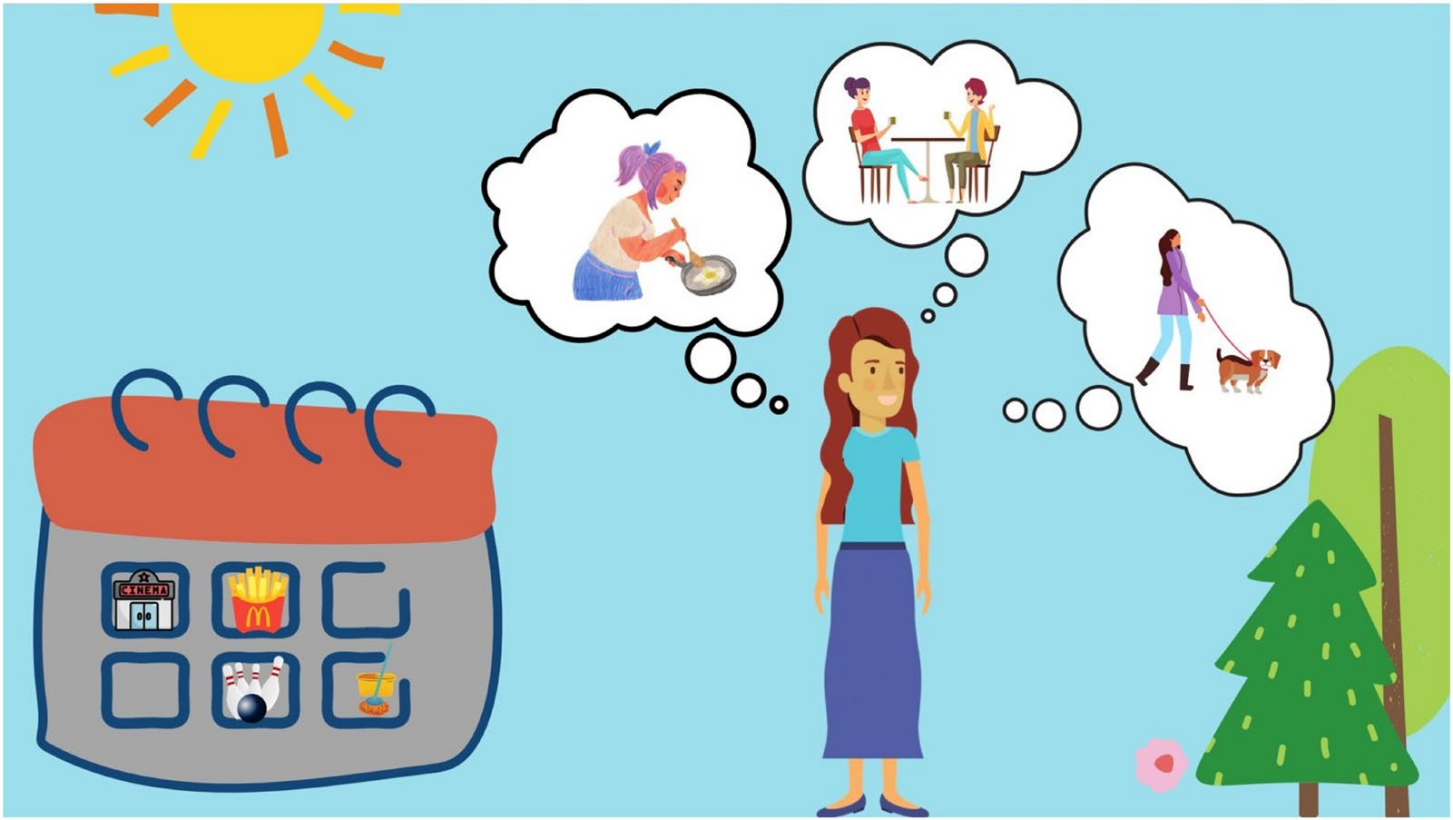
Image designed to explain the concept of capabilities.

In Session 5, the sessions addressed a core concept of the Capability Approach; capabilities. This concept was challenging to operationalise as capabilities refer to the real opportunities or freedoms people have to do things they value, rather than what they actually do. Earlier project discussions revealed confusions about Sen's use of the term ‘capabilities’ and everyday understandings of being ‘capable’ of doing something. After careful consideration, an illustration of a fictional character, Carla, was produced (Figure 2). This featured thought bubbles showing activities Carla loved doing—walking the dog, having coffee with friends and cooking—contrasted with a weekly calendar showing what activities she did which were bowling, McDonalds and the cinema. We discussed how, while Carla's life may appear full, the lack of things she valued doing meant her life was unfulfilled and even empty.

Participants were invited to share what it was they loved doing, what might stop them doing these activities and how that might feel. In this session, Jenny's thinking, supported by contributions from other participants, crystallised key insights from the learning community.

Jenny (Support manager): *I just find it, like, if I*

'

*m having a particularly stressful day, or I*

'

*ve got a really big piece of work coming in* *that*

'

*s* *quite stressful, err, or I*

'

*ve got a lot to think about when I*

'

*m driving home and I don*

'

*t want to, I put the music on and then after about 3 or 4 minutes find myself humming and singing along to it. So it must take my mind off it subconsciously, I always feel, a little bit better I suppose. […]*

*Just looking at the calendar and things, and looking at care as a whole, because it*'*s really generic, you tend to put the activities as one block thing they do in a day, that*'*s it, you know, go to McDonalds. That*'*s the end of it. Errm, but the things that people tend to enjoy, like spending time with family, or being on their own, or listening to music, they are the things that fit in around everything else, and we never seem to timetable that, or really do anything about it, but that*'*s, you know, people always seem to have one thing on their timetable to do that day, or in the morning, or in the evening and that*'*s, that is not real life is it? Really, so I think it*'*s important that we show what real life is. Even if it is waiting in for the boiler man. You know, we could be doing a lot of other things that we like to do, need to relate that as well I think, not just these one actions that we*'*re going to do for the day, like going to McDonalds in the morning, go to the park in the afternoon. That*'*s like 3 hours worth of the day, if that. […]*

Ros: *One of the things that springs to my mind is: asking those questions about when choices are documented and likes and dislikes are documented. How often, really, does that get looked at? And is it, and changed and, that kind of, that*'*s where my mind*'*s at. So yeah, to sort of start having a look, you know I*'*m quite lucky, I get to visit lots of homes and when I*'*m there, I see lots, you know, see all the folders and files of people, where, let*'*s sort of blow the dust off some of that and, and, I might be pleasantly surprised, err, hopefully I am*.

Mel: *Mmmmm*.

Research team member: *How about you Jenny, you look like you*'*re thinking, hard?*

Jenny: *Yeah, no, I*'*ve, because I*'*ve just taken over a new service, so I think I*'*m going to be looking at the accessibility calendar, so in regards to, we spoke about sort of timetables and activities, and obviously from a CQC [Care Quality Commission] point of view, we*'*ve got to document that, that people do do their things, but it*'*s how to make it accessible to the people we support, and how to capture that, you know, ten minutes in the morning singing as you go up the stairs, because that*'*s really important to me, so how I would, how do I capture that, how would I capture, there*'*s a gentleman who likes to sit outside with the wind. So how do I capture that and make sure that*'*s built in? But given the, the importance and praise that it*'*s worth, ten minutes out there is worth just as much as going to see Britney Spears to me. So it*'*s how that is highlighted and the importance, you know, CQC and external auditors come in and* “*oh, he*'*s just sitting outside, he*'*s not doing very much” and it*'*s how we show and demonstrate those little important things to them*.

Jenny in her thinking aloud here, highlighted the artificiality of care plans which prioritise timetabled, observable and ‘visible’ activities while marginalising the informal, everyday elements of ‘real life’. The compartmentalisation of time into discrete blocks leaves little space for small, meaningful pursuits that generate and sustain wellbeing. Jenny's final comment about demonstrating the importance of these ‘little important things’ to external bodies like the CQC was pivotal in deepening our collective understanding of what constitutes good support.

Jenny's hesitant articulation of what listening to Britney Spears meant to her prompted other similarly valued moments; which became known as ‘10 minutes of Britney’. It became apparent these activities, which included a cup of tea in a favourite mug, dancing in the kitchen, walking the dog, spending time with family, or watching reruns of Coronation Street on Youtube, are small, easy to organise and often free, and yet appear inconsequential within social care systems and frameworks which prioritise procedural compliance over relational and personal value.

Between 2022 and 2023, project findings were disseminated through a one‐hour ‘tour’ session (delivered online or in person) to diverse audiences, including the Care Quality Commission, NHS England, Bradford City Council, the Social Care Institute for Excellence, Lancashire and South Cumbria NHS Trust, and social care provider Community Integrated Care. Co‐facilitated by two team members with intellectual disabilities and two family carers, the sessions invited audiences to offer their own ‘10 Minutes of Britney’ examples. These mirrored those from the learning community: low‐cost, easily organised activities such as walking, baking, reading, spending time with family, smelling a dog's head or binge‐watching a box set. The tour sessions further highlighted how what people value doing changes, and people can have many ‘10 Minutes of Britney’.

## 8 Discussion

This project emerged from our frustration generated by the weight of evidence suggesting how the lives of people with intellectual disabilities could be improved, and lack of meaningful change. Following Johnson et al. (2010) we designed a project underpinned by CA working with people with intellectual disabilities and family carers. Our findings suggest that research can be based on the assumptions of researchers, influenced by the ideological and societal imposition of particular ways of being and doing. Using an approach that centred the importance of opportunities and freedoms, of what people value doing, revealed how simple, ordinary, low‐cost activities can be lost within social care settings.

Although the Flourishing Lives Learning Community was intentionally established for research purposes rather than consisting of a spontaneous grouping, participants exhibited a strong commitment to thinking together; a process identified as the most critical element of effective learning communities (Pyrko et al. 2016). The sustained rhythm and vibrancy over several months is evidenced in the extracts above, as well as the wider intensity of engagement with topics discussed. These surfaced various tensions within the context of support giving and receiving, including the prioritisation of procedural tasks over getting to know people. Not attending to the latter inevitably impinges on the development of relationships which should be key to social care support, and a priority within formal care programmes.

The growing momentum and coherence within the community became apparent as conversations around these themes progressed toward the fifth session, which centred on CA. This session produced several lightbulb moments. A particularly salient example was Jenny's realisation that the simple pleasure a person she supported derived from feeling the wind on his face was as important to him as her own enjoyment listening to Britney Spears. This insight quickly extended to her recognition that sitting outside was rarely deemed substantial enough to warrant inclusion in a care plan, revealing how such plans often erase the ‘real life’ experiences people without intellectual disabilities enjoy.

At this juncture, the value of a learning community design explicitly grounded in CA became evident. The depth of this brief yet profound exchange was enabled by cumulative thinking together across previous sessions, skilfully designed prompts and curated research materials from experienced facilitators, and sustained participant engagement. Participants valued the dedicated time and safe space to explore issues of effective support, engaging openly across diverse roles, organisations, and perspectives. The key lightbulb moments are curated here: (https://flourishinglives.mmu.ac.uk/light‐bulb‐moments/).

We know that everyday life is something we all live in, and through (Witsø and Hauger 2020), and yet the lives of people with intellectual disabilities are treated as distinct from non‐disabled people. The denial of everyday life experiences most of us enjoy with little thought is a fundamental limitation to the current organisation, delivery and oversight of social care support for people with intellectual disabilities. One mechanism through which this can occur is person‐centred planning in which the goals of people with intellectual disabilities to design their own lives are sought (McCausland et al. 2022). We suggest that putting the spotlight on the person with intellectual disabilities and not attending to the little things that are important to all of us, generates a schism which leaves people with intellectual disabilities disconnected from everyday life and community. It is useful here to turn to Durkheim's (1969) concept of individualism which concerns people in general. Johnson (2023), drawing on Durkheim's work, highlights the importance of recognising the empty space around us all—that is, common humanity—which is a core part of the moral regard we should hold for each other. As Johnson (2023, p693) suggests, ‘we must develop a cultural and material architecture of care that is sensitive to our need for moral and symbolic treatment’. We argue that interventions, like person‐centred care, mitigate against the demonstration of moral regard because, ironically, they are person rather than people‐centred.

The revelatory potential of these interactions extended beyond participants to the research team, prompting us to re‐evaluate our own experiences and appreciate the significance of such seemingly small moments in our own lives. Recognising these, and understanding how we would feel if these moments were denied to us, was a powerful way of making visible injustices experienced by people with intellectual disabilities.

This focus on small, personally meaningful and low‐cost activities appears to currently sit outside the organisation of support offered by informal and formal carers. Responses during the learning community and tour sessions were of genuine interest and enjoyment as audience members shared their ‘10 Minutes of Britney’. Embedding this initiative more formally could potentially deliver change in how people with intellectual disabilities are supported and treated, and inject enjoyment and energy into people's lives. However, formally embedding ‘10 Minutes of Britney’ within formal care programmes could lead to the stagnation of the idea and the risk of it becoming another tick box exercise. We suggest key to avoiding this, is to ensure the idea remains firmly embedded in the space around us all, in common humanity, rather than in the lives of people with intellectual disabilities.

## 9 Conclusion

Adopting CA as the underpinning framework for a social care research project, combined with a learning community design, proved an innovative and effective means of creating dedicated space and time for social care practitioners, people with intellectual disabilities, family carers, and academics to think differently together about what constitutes a good life. This approach yielded important yet straightforward insights. Informed by findings from Stage 1 of the project, the learning community sessions deliberately diminished binary distinctions between carers and those receiving care. Discussions centred on universal aspects of human flourishing—elements of everyday life that hold significance for us all.

With minor rephrasing, Jenny's question captures a fundamental concern for social care: How can we recognise and affirm the small, everyday things that matter to us all? Once these valued elements are identified, ensuring people with intellectual disabilities can access the opportunities and freedoms to pursue them becomes central to effective support.

In the context of prolonged austerity and an under‐resourced social care system, this paper's primary contribution lies in demonstrating that these personally meaningful activities, what we term ‘10 Minutes of Britney’, are typically low‐cost and straightforward to facilitate. Prioritising such capabilities offers a practical, resource‐efficient pathway to enhancing wellbeing and promoting more equitable support. However, it is key that these considerations remain relevant to us all, rather than a bespoke intervention for people with intellectual disabilities.

## Funding

The research is funded by the National Institute for Health and Care Research (NIHR) under its School for Social Care Research programme. The views expressed are those of the authors, and not necessarily those of the NHS, the NIHR or the Department of Health and Social Care.

## Ethics Statement

Approved by the Manchester Metropolitan University Research Ethics and Governance Committee.

## Consent

The authors have nothing to report.

## Conflicts of Interest

The authors declare no conflicts of interest.

## Data Availability

The data that support the findings of this study are available on request from the corresponding author. The data are not publicly available due to privacy or ethical restrictions.
